# Laparoscopic Management of Hepatic Abscess From Ingested Chicken Bone

**DOI:** 10.7759/cureus.13403

**Published:** 2021-02-17

**Authors:** Jai W Hoff, George Castrisos, Goutham Sivasuthan, Clay Renwick

**Affiliations:** 1 General Surgery, Rockhampton Hospital, Rockhampton, AUS

**Keywords:** laparoscopic surgery, hepatic abscess, ingested foreign body, gastro-intestinal perforation, general surgery

## Abstract

A 68-year-old man presented to the hospital with severe right upper abdominal pain, fevers, nausea and lethargy. He deteriorated into septic shock and was found to have a hepatic abscess on computer tomography imaging. After multiple investigations and continual deterioration, he underwent an exploratory laparoscopy which revealed a chicken bone within the liver parenchyma resulting in a large hepatic abscess. The patient required a second laparoscopic washout and prolonged antibiotics, subsequently recovering well. This rare case highlights the difficulty in diagnosing hepatic abscesses caused by gastrointestinal foreign bodies, and successful management with laparoscopic surgery.

## Introduction

Accidental ingestion of foreign bodies is not uncommon, with the majority passing through the gastrointestinal tract without complication [1]. Less than 1% of ingested foreign bodies cause perforation within the gastrointestinal tract [2]. Presentation is often delayed due to an insidious onset of symptoms, and incongruent patient histories. Diagnosis is frequently based on computed tomography (CT) or ultrasonography, modalities that are sensitive but not specific [3-4]. An ingested foreign body should be considered for all hepatic abscess without a clear precipitant. There should be a low threshold to expediting surgery with the evidence suggesting improved patient outcomes with laparoscopic surgery compared to open procedures.

## Case presentation

A 68-year-old man presented to a remote hospital with severe right upper quadrant abdominal pain. His other symptoms included subjective fevers, nausea, anorexia and lethargy for two days. He denied altered bowel habits, overseas travel or ingested any foreign bodies. Prior to this admission, he was a gentleman with a past medical history of gastro-oesphageal reflux disease, peripheral neuropathy and benign prostatic hypertrophy. 

On physical examination, he was peritonitic with rebound tenderness and rigidity. Within hours of presentation, he became septic with fevers, tachypnea, hypotension and a new oxygen requirement. He was started on empirical intravenous (IV) antibiotics, received fluid resuscitation and sent for CT scan of the abdomen. The patient was transferred to the nearest regional hospital and admitted to intensive care under the general surgeons.

**Figure 1:**
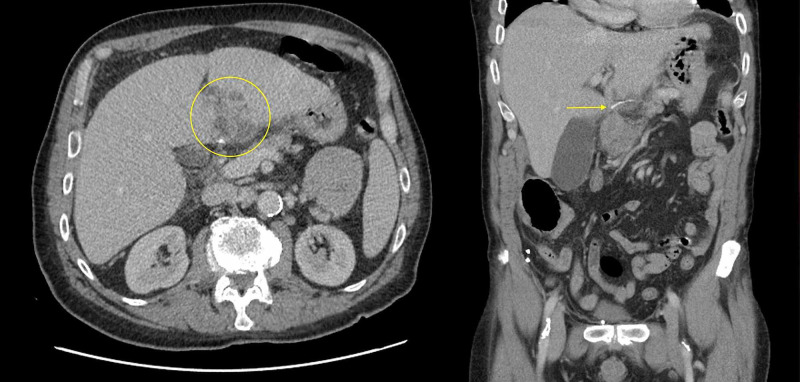
CT scan demonstrating the hepatic abscess with an associated linear density and localised inflammation changes CT - Computed tomography

His bloods on presentation showed an inflammatory/infective process (white cell count 18.3 x 10^9^/L, C-reactive protein 251 mg/L), acute kidney injury (creatinine 140 μmol/L) and mildly deranged liver function tests (bilirubin 16 μmol/L, ALP 134 U/L, GGT 85 U/L, ALT 66 U/L, AST 45 U/L, LD 277 U/L). The CT scan summary described a likely hepatic abscess (5.4x4.9x4.0cm, neoplastic lesion not excluded) and a moderate wall thickening involving an adjacent short segment of the mid transverse colon with surrounding oedema and fluid likely representing colitis (Figure 1). A linear hyperdense material adjacent to the hepatic lesion was described in the body of the report.

In view of the CT findings, a diagnostic flexible sigmoidoscopy was performed, which was unable to identify any significant mucosal abnormalities within the transverse colon. Subsequently, a liver ultrasound was done, suggesting a new hepatic extracapsular fluid collection had replaced the hepatic abscess demonstrated on CT. Imaging guided drainage was requested, however, not attempted due to poor visualization of the area. 

**Figure 2:**
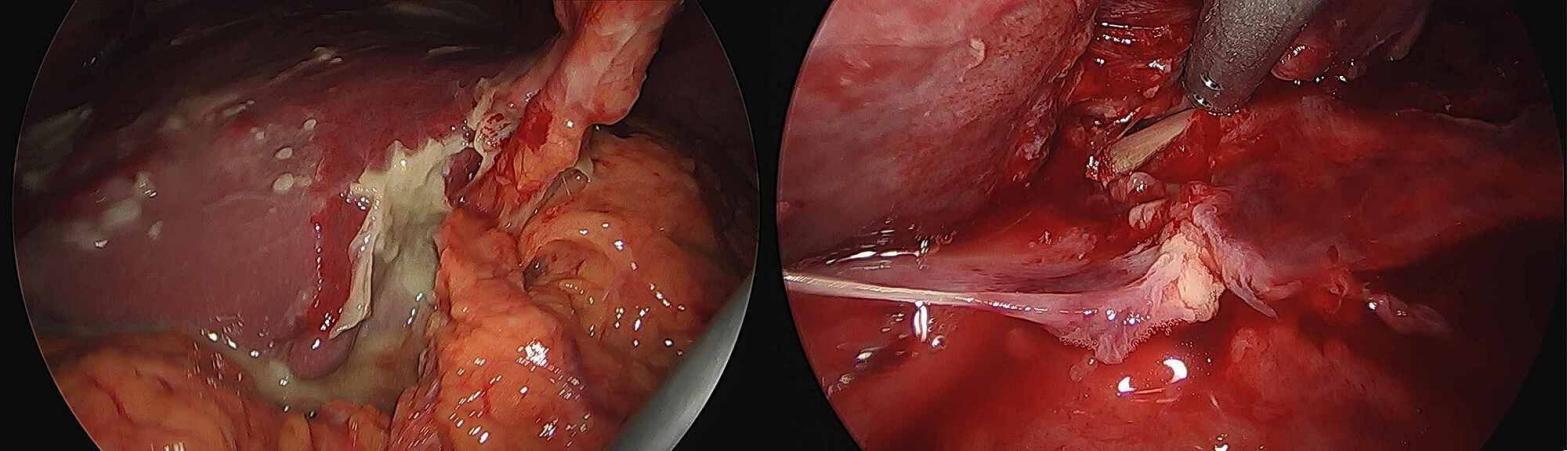
Intraoperative findings of pus at the posterior aspect of the liver. Following blunt dissection, a chicken bone was found extruding from the liver parenchyma

The patient deteriorated with increasing inflammatory markers and new-onset atrial fibrillation despite being on IV antibiotics. A discussion with the second radiologist regarding the original CT abdomen suggested the scan abnormalities may represent a duodenal perforation with a foreign body corresponding to the hyperdense lesion. An exploratory laparoscopy was performed which revealed a sealed duodenal perforation with pus surrounding the posterior aspect of the liver (Figure 2). Further dissection led to the discovery of a chicken bone being identified within the caudate lobe of the liver.

The bone was removed laparoscopically without complication. Over the following three days, the patient progressed slowly and a follow-up CT abdomen showed a persistent hepatic collection. The patient underwent a repeat laparoscopic washout and with a drain inserted. The pus culture grew streptococcus intermedius and antibiotics were rationalized accordingly. The patient showed good clinical improvement following the second washout. The abdominal drain was removed on day 4 and the patient was discharged on a prolonged course of oral antibiotics.

A follow-up scan at one-month post-discharge showed complete resolution of the hepatic abscess. A follow-up gastroscopy at three months showed some mucosal scarring in the duodenum but was otherwise unremarkable. During subsequent follow-up, the patient felt that it took him 12 months to make a full recovery.

## Discussion

Accidental ingestion of foreign bodies is not uncommon, with the majority passing through the gastrointestinal tract without complication [1]. Less than 1% of ingested foreign bodies cause perforation within the gastrointestinal tract [2]. Hepatic abscesses are most commonly caused by biliary pathology, followed by portal seeding, arterial seeding and lastly, penetrating trauma. Hepatic abscess from gastrointestinal foreign bodies is rare occurrences. The most common causes of hepatic abscesses from foreign bodies include fish bones, followed by toothpicks and chicken bones [1]. 

Often patients do not recall ingesting foreign objects, being consumed frequently within a food bolus, days/weeks prior to presentation. A diagnosis is often suggested after radiological identification of an abscess; however, these findings are often non-specific with respect to underlying aetiology [3]. A systematic review found CT was better than ultrasound in identifying the offending foreign body; however, the accuracy was low (55% vs 27%) [4].

When a foreign body peroration is suspected, surgical intervention is suggested as source control, with conservative management only being effective 9.5% of the time [4]. If the foreign body is suspected to still be within the wall of the intestinal tract, the foreign body can be attempted to be removed endoscopically [5]. A previous literature review found 61% of foreign body removal was done via laparotomy as compared to 9% done laparoscopically [6]. If the conditions are favourable, the foreign body associated hepatic abscesses can be managed with laparoscopic surgery, leading to reduced morbidity and an expedient recovery [7].

This particular case is rare as the chicken bone caused sealed perforated of the duodenum, lodging itself within the caudate lobe of the liver. The case highlights challenges involved with making a diagnosis, as the patient’s history was unremarkable and imaging was difficult to interpret. The patient continued to deteriorate despite broad-spectrum antibiotics; hence, swift surgical management was implemented. This case demonstrated successful management of the hepatic abscess with laparoscopic surgery, which led to the patient having a full recovery.

## Conclusions

Ingested foreign bodies should be considered for all hepatic abscess without a clear precipitant. It is important to note that patients who have complications from ingested foreign bodies can present much later than the initial event, making it difficult to make a diagnosis of history. Diagnosis of foreign bodies causing perforation is often made on ultrasound and CT imaging, however, can be difficult to interpret and foreign bodies often cannot be accurately identified. There should be a low threshold to have surgical intervention as many patients can be successfully treated with laparoscopic surgery.
